# Investigating potential sand fly vectors after the first reported outbreak of cutaneous leishmaniasis in Ghana

**DOI:** 10.1186/s13071-023-05767-4

**Published:** 2023-04-28

**Authors:** Dziedzom K. de Souza, Seth Offei Addo, Kwame Desewu, Chukwunonso O. Nzelu, Mba T. Mosore, Shirley Nimo-Paintsil, Naiki Attram, Maxwell Appawu, Michael D. Wilson, Daniel A. Boakye

**Affiliations:** 1 Noguchi Memorial Institute for Medical Research, College of Health Sciences, University of Ghana, LG 581, Legon, Accra Ghana; 2 Ghana Health Service, Accra, Ghana; 3 Snyder Institute for Chronic Diseases, Departments of Microbiology, Immunology, and Infectious Diseases, Cumming School of Medicine and Comparative Biology and Experimental Medicine, Faculty of Veterinary Medicine, University of Calgary, Calgary, Canada

**Keywords:** Cutaneous leishmaniasis, Ghana, Neglected tropical disease, *Phlebotomus*, *Sergentomyia*

## Abstract

**Background:**

Leishmaniasis is a parasitic disease caused by species of the genus *Leishmania*, which are transmitted through the bite of infected female sand flies. Since the first reported outbreak of cutaneous leishmaniasis in Ghana, in 1999, there has been limited published information on its vectors and reservoir hosts there. Previous studies have shown strong dominance of the sand fly genus *Sergentomyia* over the genus *Phlebotomus* in Ghana. Thus the aim of this study was to determine the possible sand fly vector species in Ghana, as well as their human-feeding behavior, from the time of the first reported outbreak of CL in the country.

**Methods:**

Sand flies were collected from randomly selected houses in three communities. They were identified and used for blood meal source identification and the detection of *Leishmania* infection using molecular methods.

**Results:**

A total of 1051 female sand flies were morphologically identified, of which *Sergentomyia africana africana* (29%) was the predominant species. Among the 275 female sand flies that had blood-fed, the identified blood meal sources included chicken (33.8%) and goat (12.4%); the percentage of human blood meals was 32%. Single-source and mixed-source blood meals were identified in *Sergentomyia africana africana* (11.6%), *Sergentomyia ingrami* (14.9%) and *Sergentomyia simillima* (20%), with *S. simillima* having the highest proportion of blood meals that included human blood (14.6%). Using molecular methods, unfed sand flies and identified human-feeding species were examined for the presence of *Leishmania* DNA. Pool screening analysis revealed three pools of *S. ingrami* positive for *Leishmania major* DNA, with an infection rate of 1.27% (95% confidence interval 2.467–3.647).

**Conclusions:**

The findings suggest that some *Sergentomyia* species may be involved in the transmission of cutaneous leishmaniasis in Ghana. However, the role of *S. ingrami* as a vector of leishmaniasis in Ghana needs to be conclusively validated by isolating the parasite from this species and through experimental transmission studies.

**Graphical abstract:**

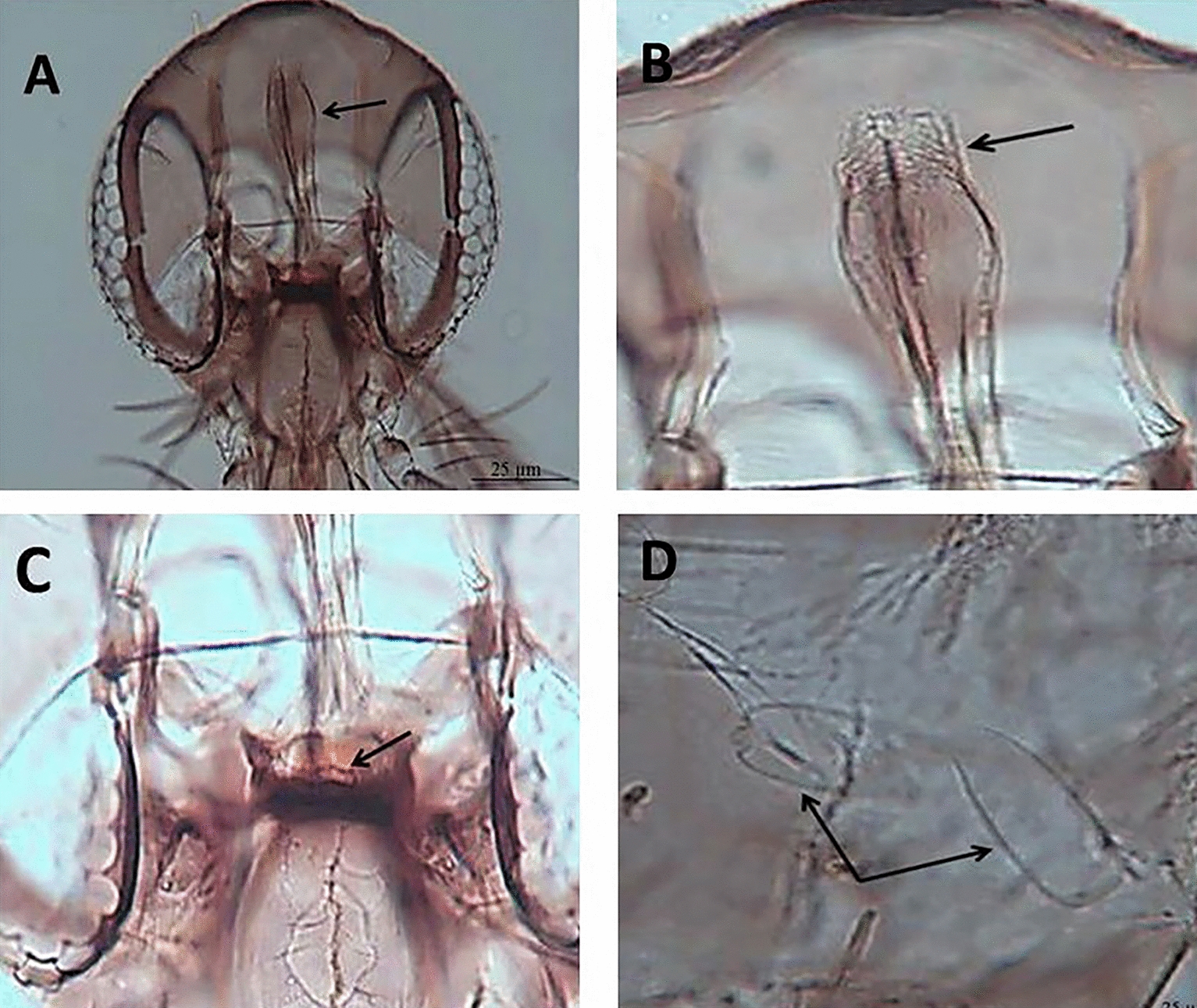

**Supplementary Information:**

The online version contains supplementary material available at 10.1186/s13071-023-05767-4.

## Background

The World Health Organization estimates that, worldwide, 98 countries are endemic for leishmaniasis. In Africa, countries endemic for the disease are mostly located in the Northern, Central and Eastern Africa subregions, the last of which includes the Horn of Africa. A cutaneous leishmaniasis (CL) belt runs from Mauritania, Gambia and Senegal in the west of Western Africa to Nigeria, in the east of the subregion, and its bordering country, Cameroon, which is in Central Africa. However, leishmaniasis appears to be one of the least recognized or underreported parasitic infections in Western Africa [1]. Northern Ghana is also found within the CL belt, yet there had been no reports of the disease in that area, or anywhere in the country, until 1999, when an outbreak was reported from three sub-districts in southeastern Ghana, Ho/Shia, Kpedze/Vane and Tsito in the Ho District, which borders Togo. A total of 2348 cases before 2003 and 6185 cases between 2003 and October 2004 were reported during the outbreak [2]. Urgent investigations were undertaken to understand the outbreak in this atypical ecocline for CL. The initial studies identified the parasite as *Leishmania major* [3], but a follow-up study indicated that it was a new species [4], which was subsequently confirmed and the species named *Leishmania* (*Mundinia*) *chancei* [5]. The studies undertaken using real-time polymerase chain reaction (PCR) analysis aimed at identifying the reservoirs among small mammals and local livestock did not reveal the presence of *Leishmania* DNA. Therefore, the etiology and reservoirs of the leishmaniasis outbreak remain unknown [6].

Old World CL is reported to be transmitted by over 40 species of phlebotomine sand flies (Diptera: Psychodidae) [7]. Several species that belong to two genera of phlebotomine sand flies, *Phlebotomus* and *Sergentomyia*, have been reported from West Africa, but only *Phlebotomus duboscqi* has been implicated as a vector of CL there [8]. *Sergentomyia* species are not usually thought to transmit human leishmaniasis, although Mukherjee et al*.* [9] reported the detection of *Leishmania* DNA in *Sergentomyia babu* during an epidemic of visceral leishmaniasis in India, Mutinga et al. [10] found a natural infection of *L. major* in *Sergentomyia garnhami* in Kenya, and Maia et al.[11] discussed the potential role of *Sergentomyia* species in the transmission of *Leishmania*.

Determining the vectors of CL in the outbreak area is important in the planning of effective control strategies. Entomological studies undertaken from 2004 until 2008 indicated that almost 99.5% of the sand fly species in Ghana belonged to the genus *Sergentomyia*, while *Phlebotomus rodhaini* accounted for the remaining 0.5% (Additional file 2: Table S1). The density of the latter was so low that its vectorial role was uncertain [1]. Although *Sergentomyia* species predominated in the collections, they are not considered vectors of human leishmaniasis. Thus, to determine the possible vector species in the outbreak area, we collected sand flies from human habitations and analyzed the blood meals of the blood-fed females to identify the human-feeding species. However, since human-feeding behavior of an insect species is insufficient to implicate it in disease transmission, the criteria for vector incrimination [12, 13] included examining the species that fed on humans for infection with *Leishmania* species as found in the human hosts [12, 13]. Herein, we report the detection and identification of *L. major* DNA in human-feeding *Sergentomyia* through molecular methods.

## Methods

### Sand fly sampling and preservation

Ghana is bordered by Burkina Faso to the north, the Gulf of Guinea to the south, Togo to the east and Cote d'Ivoire (Ivory Coast) to the west. Prior to the outbreak of CL in Ghana in 1999, an outbreak occurred in Burkina Faso, in 1996, with about 1845 cases reported [14]. No CL cases were reported from the other neighboring countries of Ghana, and the outbreak region was close to the southern part of Togo.

In the present study, sand flies were collected in August, September and November 2007 (wet season) from three outbreak communities: Klefe (06º37ʹN–00º26E), Taviefe (06º66ʹN–00º47ʹE) and Hlefe (06º42ʹN–00º22ʹE). These sites are within Ho Municipal District, where the ecosystem is semi-deciduous forest. The mean temperature in the municipality ranges from 22.0 to 32.0 °C, and it has a wet and dry season. A significant number of inhabitants are engaged in farming, which is the main source of income. In the communities, the most commonly found domestic animals are small ruminants and poultry. In earlier, unpublished studies monthly collections were undertaken using sticky traps and CDC light traps baited with carbon dioxide to collect indoor and outdoor resting sand flies. In those studies, *Sergentomyia* species were the most commonly detected species (Additional file 2: Table S1). However, in the present study, the focus is on indoor resting sand flies. Five houses were randomly selected in each of the three communities and sand flies were aspirated at varying heights from the walls of three rooms in each house. The sand flies were found mostly in dark and obscure locations, thus torchlight was used to locate them. A battery-powered hand-held aspirator was used for their collection. Sampling was carried out from 0630 to 0830 hours and 1630–1830 hours over 5 days per month. The collected sand flies were euthanized with dry ice and preserved dry on silica gel in 1.5-ml Eppendorf tubes.

### Sand fly dissection and identification

The sand flies were sorted into blood-fed and non-blood-fed females, which were processed differently. The blood-fed females were used for blood meal analysis to determine their human-feeding behavior. The non-blood-fed females were kept for future investigation.

The head and the last three posterior abdominal segments of the dried blood-fed females were detached using sterile dissecting pins and placed on labeled slides in distilled water. The blood-engorged middle section of the abdomen of each sand fly was labeled identically to the head and posterior abdominal segments, kept dry on silica gel, and stored until processed for the determination of the blood meal source(s).

Approximately two drops of clearing medium (1:1, chloral hydrate:phenol) were applied to the head and posterior abdomen on a slide for clearing of the soft tissue over an 8–12 h incubation. A cover slip smeared with vaseline at the edges was placed on top of the specimen to seal the cover slip to the slide and prevent the volatile clearing medium from leaking or drying out. The specimen was then incubated overnight to clear the remaining tissue and chitin. The cover slip was then carefully removed and mounting medium (10:8:70:5:3, distilled water:gum arabic:chloral hydrate:glycerin:acetic acid) was added. The parts were allowed to incubate on a heating plate at 40 °C for 3 weeks and were then observed under a phase contrast microscope for species identification using taxonomic keys [15].

### Identification of blood meal sources and analysis

Blood meal origin was determined by direct enzyme-linked immunosorbent assay (ELISA), using anti-human immunoglobulin (Ig) G, anti-goat IgG, anti-bovine IgG and anti-chicken IgG with standard methods [16, 17]. The human blood index (HBI) was calculated for the communities, and also for months, as per the definition of Lardeux et al*.* [18], based on the number of samples of each species where the blood was derived from humans, and the total number of samples analyzed.

### DNA extraction

Unfed sand flies collected at the same time as the blood-fed flies, as well as the carcasses of the human blood-fed ones, were used to determine natural infections. The dissected thorax and attached proximal abdomen of unfed sand flies and carcasses of human blood-fed ones were separated according to species and collection site and pooled into groups of 10 individuals in sterile tubes for DNA extraction. Pools of 10 flies were made to maximize the possibility of detecting flies positive for *Leishmania* because pools comprising material from more flies sometimes result in PCR inhibition due to the presence of PCR inhibitors in the insect cuticle. Total genomic DNA from each pool of female sand flies was extracted using the Qiagen DNA Mini Kit (Qiagen, Valencia, CA) as per the manufacturer’s instructions. DNA was eluted in 50 µL of elution buffer and stored at − 20 °C until required.

### Detection and identification of *Leishmania* species

PCR reactions targeting internal transcribed spacer region 1 (ITS1) were performed for the detection of *Leishmania* DNA in sand flies. The primer sequences were 5ʹ-TGATACCACTTATCGCACTT-3ʹ (L5.8S) and 5ʹ-CTGGATCATTTTCCGATG-3ʹ (LITSR) [19]. Amplification reactions were carried out in 25-µl volumes containing 200 µM of dNTP, 1.5 mM MgCl_2_, two units Taq polymerase, 500 nM of each primer and 0.5 µl of extracted DNA as a template. Each PCR reaction included 5 µg positive control DNA [from reference strains *Leishmania major* (IPAP/EG/89/S1-177) and *Leishmania* *tropica* (WR664)] and a negative control (water). After initial denaturation at 95 °C for 2 min, PCR amplification was performed with 34 cycles consisting of denaturation (95 °C for 20 s), annealing (53 °C for 30 s), and extension (72 °C for 1 min), followed by a final extension cycle at 72 °C for 6 min. Five microliters of the amplification reaction product was resolved on a 2% agarose gel and visualized.

ITS1–PCR products showing a *Leishmania*-specific band on agarose gel were digested with HaeIII (Invitrogen) for species identification [20]. Restriction fragments were subjected to electrophoresis on 2% agarose gel and compared with reference DNA of *L. major* and* L*. *tropica*.

### Statistical analysis

The infection rate of *Leishmania* parasites in indoor-captured sand flies was estimated using the PoolScreen2 program generously provided by Dr Charles Katholi (The University of Alabama, Birmingham) [21]. The algorithm was used to calculate the maximum likelihood estimate of *Leishmania* infection in sand flies, with a 95% confidence interval.

### Ethics statement

The study did not involve the collection of human samples or the direct participation of community members. It was focused only on the collection of sand flies resting in houses. Thus, the communities where sampling was done were informed of the project and consent was sought from the local authorities of each community. Informed oral consent was obtained from the households where sand fly sampling was carried out. Due to the high illiteracy rate in the communities, the acceptance of the heads of households was recorded by the team leader. The data were collected in such a way that the results could not be linked to individuals, or households, thus ensuring their anonymity, and approval was obtained from the Institutional Review Board of the Noguchi Memorial Institute for Medical Research.

## Results

A total of 1845 indoor-resting sand flies were collected from human dwellings in the three focal outbreak communities during the collection period, from August through September 2007. The highest number of flies, 916 (50%), was collected from Klefe, followed by 645 (35%) from Hlefi and 284 (15%) from Taviefe. The total number of females and males was 1342 and 503, respectively, without significant differences in the female:male ratio observed among the collections from the different communities.

### Morphological identification of sand flies

Of the 1342 female sand flies captured, 1051 were morphologically identified to species level. Nine sand fly species were identified, all of which belonged to the genus *Sergentomyia* (Table 1)*. Sergentomyia africana africana*, *Sergentomyia ingrami*, *Sergentomyia dissimillima* and *Sergentomyia simillima* were the most abundant species in all the study areas, with a cumulative prevalence of 29.0%, 24.4%, 18.8% and 21.5%, respectively (Table 1). Of the 1342 total captured females, 275 (20.5%) were found to have taken a blood meal (Klefe, *n* = 175; Hlefi, *n* = 65; Taviefe, *n* = 35) (Table 2). Photomicrographs of the morphological features of female *S*. *ingrami* are provided (Additional file 1: Fig. S1) showing the pharynx (Additional file 1: Fig. S1A), pharyngeal armature [magnified in section 1A (Additional file 1: Fig. S1B)], cibarium (Additional file 1: Fig. S1C) and spermatheca (Additional file 1: Fig. S1D).

**Table 1:** Female phlebotomine sand flies collected from human habitations in each of the three outbreak communities of Ho District, Ghana

Species (subgenus)	Sand fly collection location [*n* (%)]			Total
	Taviefe	Klefe	Hlefi	
*Sergentomyia africana africana*	40 (3.8)	70 (6.7)	195 (18.6)	305 (29.0)
*Sergentomyia* (*Neophlebotomus*) *ingrami*	56 (5.3)	120 (11.4)	80 (7.6)	256 (24.4)
*Sergentomyia* (*Sintonius*) *clydei*	0 (0.0)	0 (0.0)	1 (0.1)	1 (0.1)
*Sergentomyia* (*Sergentomyia*) *buxtoni*	0 (0.0)	0 (0.0)	1 (0.1)	1 (0.1)
*Sergentomyia* (*Sergentomyia*) *simillima*	31 (2.9)	140 (13.3)	55 (5.2)	226 (21.5)
*Sergentomyia* (*Sergentomyia*) *schwetzi*	7 (0.7)	0 (0.0)	0 (0.0)	7 (0.7)
*Sergentomyia* (*Sergentomyia*) *hamoni*	2 (0.2)	50 (4.8)	4 (0.4)	56 (5.3)
*Sergentomyia* (*Sergentomyia*) *distinct*	0 (0.0)	1 (0.1)	0 (0.0)	1 (0.1)
*Sergentomyia* (*Sergentomyia*) *dissimillima*	16 (1.5)	134 (12.7)	48 (4.6)	198 (18.8)
Total	145 (13.8)	515 (49.0)	391 (37.2)	1051 (100)

**Table 2:** Proportions of blood-fed individuals of sand fly species collected in each of three outbreak communities of Ho District, Ghana

Community	Sand fly species				Total
	*Sergentomyia africana africana*	*Sergentomyia antennata*	*Sergentomyia ingrami*	*Sergentomyia simillima*	
Hlefi	27/74 (36.5%)	0/1 (0.0%)	21/95 (22.1%)	17/105 (16.2%)	65
Klefe	43/74 (58.1%)	1/1(100%)	44/95 (46.3%)	87/105 (82.9%)	175
Taviefe	4/74 (5.4%)	0/1 (0.0%)	30/95 (31.6%)	1/105 (0.9%)	35
Total	74	1	95	105	275

### Identification of sand flies and their blood meal sources

Two hundred and seventy-five blood-fed females were dissected and successfully identified. Fifteen other flies that had also taken a blood meal were damaged during processing and could not be identified definitively. All 275 blood-fed individuals belonged to one of four species of the genus *Sergentomyia*—*Sergentomyia simillima*, *Sergentomyia ingrami*, *Sergentomyia africana africana* and *Sergentomyia antennata*—in the following proportions: 38.2%, 34.5%, 26.9% and 0.4%, respectively (Table 2).

The 275 blood-engorged identified female sand flies were tested for the origin of their blood meal using direct ELISA. Of these, 128 (46.6%) had gut contents that were reactive to at least one of the three antibodies used for this investigation. The remaining blood-fed sand flies had possibly fed on animals other than those tested for in this study. Reactive gut contents of 66 of the sand flies indicated patent mixed blood meals (Table 3). Thus, the majority of the blood-fed females (24.0%, *n* = 66) with positive reactive gut content had taken a multiple blood meal (two or three sources), whilst the remaining 22.6% (*n* = 62) had taken a single blood meal. Of the multiple blood meals, 95.5% (63/66) included human blood, and 40.3% (25/62) of the single-source blood meals comprised human blood. None of the blood-fed sand flies had fed on bovine blood.

**Table 3:** Number of individuals of four species of sand flies that fed on single or multiple blood sources

Blood meal	Sand fly species				Total
	*Sergentomyia africana africana*	*Sergentomyia ingrami*	*Sergentomyia simillima*	*Sergentomyia antennata*	
Chicken	13	6	13	–	32
Human	7	9	9	–	25
Goat	0	4	1	–	5
Chicken and goat	1	1	1	–	3
Chicken and human	9	11	17	–	37
Goat and human	0	3	2	–	5
Chicken, goat and human	2	7	12	–	21
Bovine	0	0	0	–	0
Unidentified blood source	42	54	50	1	147
Total	74	95	105	1	275

**Fig. 1:**
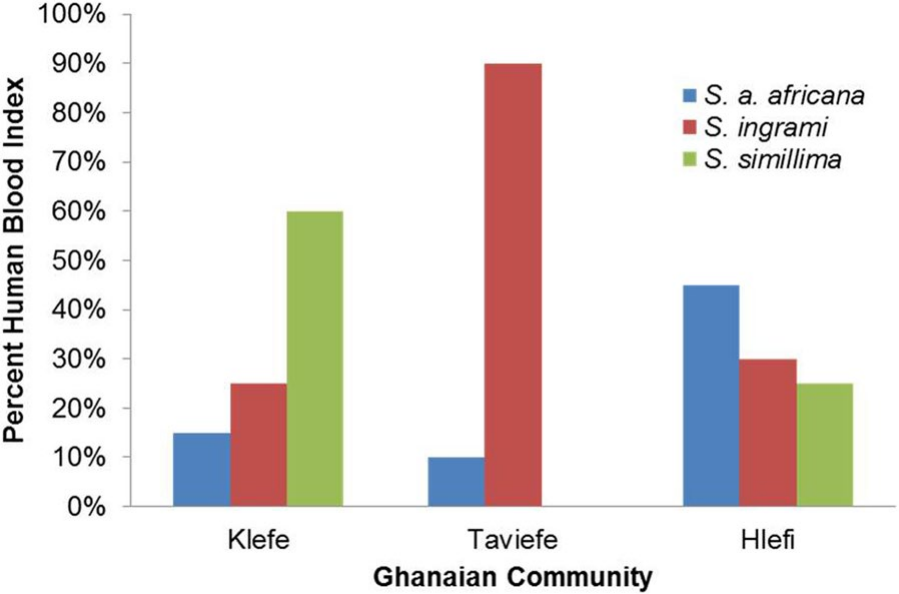
Variation in the human blood index among the collected sand fly species

Chickens were the most common source of the blood meals. However, a high proportion of each sand fly species also fed on humans. Of the sand flies analyzed, 31.6% (30/95) of *S. ingrami*, 38.1% (40/105) of *S. simillima* and 24.3% (18/74) of *S. africana africana* fed on humans. The HBI varied from community to community for each sand fly species (Fig. 1). The overall HBI for the three communities combined ranged from 0 to 31%, and the highest HBI was for *S. simillima* (Table 4).

**Table 4:** Human blood index of blood-fed females of the sand fly species collected indoors in the three Ghanaian outbreak communities

Sand fly species	Human blood index (%)
*Sergentomyia africana africana*	6.6
*Sergentomyia ingrami*	11.0
*Sergentomyia simillima*	14.6
*Sergentomyia antennata*	0

### Detection of *Leishmania* infection in sand flies

**Fig. 2:**
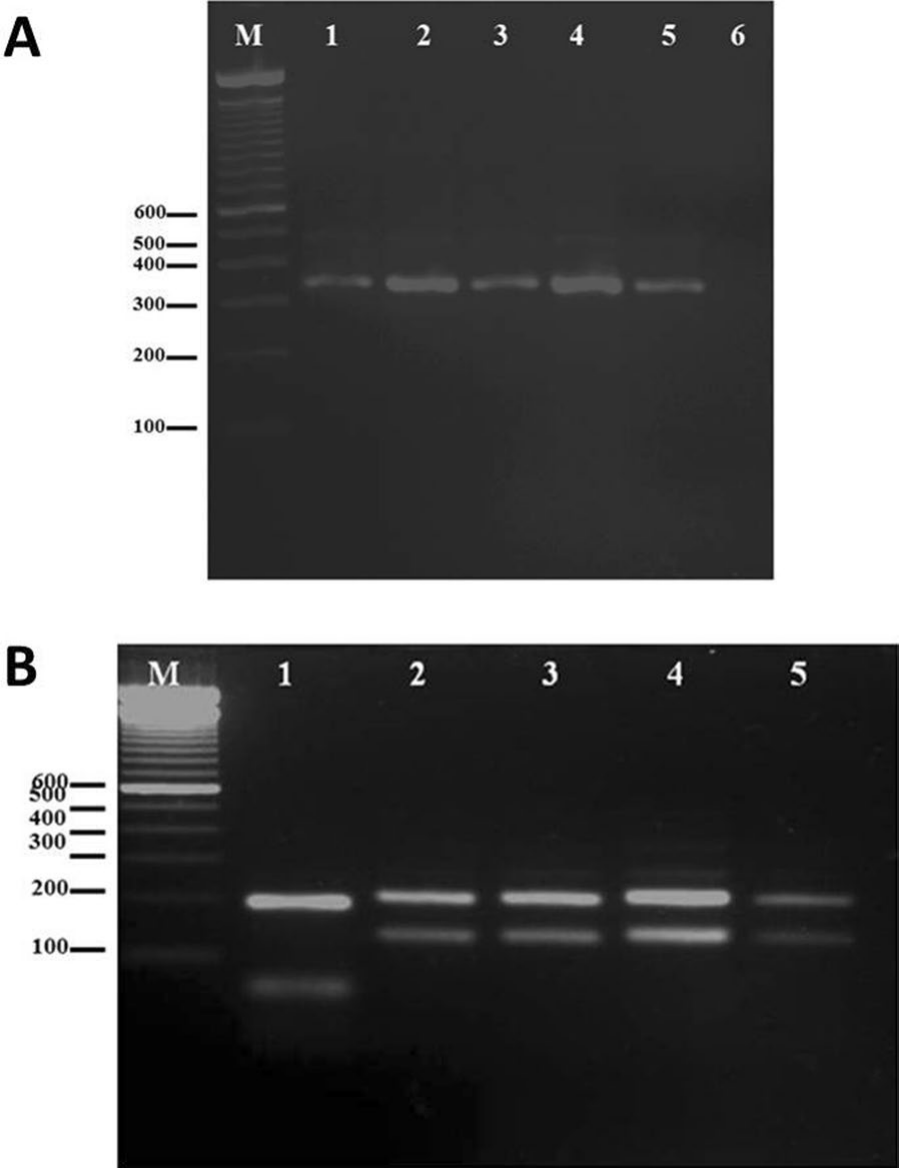
**a** Detection of *Leishmania* DNA in *Sergentomyia* *ingrami* pools using internal transcribed spacer region 1-polymerase chain reaction.* M* 100-base pair marker,* lane 1* positive control with 5 µg DNA from *Leishmania* *tropica*, *lane 2* positive control with 5 µg DNA from *Leishmania major*,* lane 3* amplified *Leishmania* DNA within the human blood-fed *S*. *ingrami* pool,* lanes *4 and 5 amplified *Leishmania* DNA within unfed *S*. *ingrami* pools,* lane 6* negative control. **b** Polymerase chain reaction-restriction fragment length polymorphism analysis of internal transcribed spacer region 1 genes obtained with HaeIII.* M* 100-base pair marker,* lane 1* *L. tropica* reference strain,* lane 2* *L. major* reference strain,* lanes 3–5* *S. ingrami* pools

Thirty pools of *S. africana africana*, 25 pools of *S. ingrami*, 19 pools of *S. dissimillima*, 23 pools of *S. simillima* and five pools of *S. hamoni* (each pool comprising material from 10 insects) were processed using PCR for *Leishmania* detection. ITS1-PCR produced a single distinct amplicon of approximately 340 base pairs in one pool of human blood-fed *S. ingrami* from Klefe and two pools of unfed *S. ingrami* from Hlefi (Fig. 2a). The PCR–restriction fragment length polymorphism analysis with endonuclease HaeIII produced fragments characteristic of *L. major* (Fig. 2B).

The minimum sand fly infection rate, assuming that each PCR-positive pool included at least one infected insect, was 0.29% among the analyzed samples. The PoolScreen2 algorithm estimated an *S. ingrami* infection rate of 1.27% (95% confidence interval, 2.467–3.647).

## Discussion

Before 1999, Ghana was not known to be endemic for CL. The outbreak of CL in Volta Region in 1999 indicated an urgent need to understand the transmission dynamics of the disease in Ghana. Monthly entomological sampling carried out from January 2006 to May 2007 revealed that 99.8% (21,980/22,016) of the sand flies collected were *Sergentomyia* and 0.2% (36/22,016) *Phlebotomus*, the putative vector of CL in the Old World (Additional file 2: Table S1). However, the density of *Phlebotomus* was so low that its vectorial role was uncertain. In the present study, only *Sergentomyia* species were identified from the sampled communities.

Human-feeding behavior is one of the essential factors for the implication of a vector in the transmission of pathogens to humans, while data on attraction to other hosts can also shed light on the association between potential vectors and natural reservoirs of pathogens. Generally, human landing catches (HLC) provide evidence of the human-feeding nature of vectors. However, the HLC method is labor intensive and also challenging in the light of ethical constraints. An alternative method for determining human-feeding behavior is to identify human blood in blood-fed insects. Accurate knowledge of the blood meal source provides critical information on host preference including human-feeding behavior under natural conditions.

In the area of the leishmaniasis outbreak in Ghana, of which the vectors were unknown, sand fly collection indicated that *Sergentomyia* species were the most predominant and *Phlebotomus* species the most insignificant species. This led to the hypothesis that *Sergentomyia* species may play a role in the transmission of leishmaniasis in this area of deciduous forest [1]. The finding in the present study that all the sand flies collected from human dwellings, including those that were blood-fed, were of the genus *Sergentomyia* is consistent with the results of previous sampling of sand flies in the same areas [2, 3], and indicates that some species of this genus do have an association with humans in the outbreak area. The genus *Sergentomyia* also predominates in other West African countries, such as Mali [22], Senegal [23] and Nigeria [24].

The proportion of sand flies that had fed on multiple blood sources was greater than the proportion that had taken a single blood meal and was indicative of opportunistic or eclectic feeding habits [25–27]. The results indicated that most of the sand flies in the area feed on any available host that is close to them, and may also engage in selecting the best food source from the range of available hosts. Moreover, the finding that about 95.5% of the multiple blood meals and 40.3% of the single blood meals originated from human hosts indicated that the *Sergentomyia* species present in the studied areas exhibit human-feeding behavior.

The sand flies had fed more on chicken blood than on human blood, which is suggestive of exophagic and endophilic behaviors. It has been reported that chicken blood supports the development of *Leishmania* in vectors. One study reported that chicken blood likely supports the development of *Leishmania mexicana* in *Lutzomyia longipalpis* [28]. Also, chicken blood was found to support the development of *L. major* in *P. duboscqi* [29]. Noguera et al. [30] observed that chicken blood enhanced the reproductive potential of sand flies, since it was quickly digested, resulted in the longest period of oviposition, the greatest number of eggs retained, and the greatest fecundity, compared with the other sources of blood, which included horses, dogs, cows, goats, pigs and humans. While the blood meal sources of 147 flies could not be identified in the present study, these may have provided some indication of the reservoirs of *Leishmania* in the study area, and this needs to be investigated further. It is also important to determine the effects of chicken blood on the transmission of *Leishmania* in Ghana.

The calculated HBI varied between sand fly species and within the same species from one locality to another. This indicates that these species do not strictly feed on humans but rather do so opportunistically, depending on the proportions of humans and other potential hosts that live in these areas and the availability of these hosts [31, 32]. The actual anthropophily of these sand flies needs to be examined by undertaking studies aimed at estimating their relative host abundance (number and mass) around the sampling sites and their preference for humans over other available hosts.

Molecular techniques (PCR) have been employed to screen sand flies for *Leishmania* infection [33–36], and represent a faster diagnostic tool than digestive tract dissection and microscopic examination. Thus, we used molecular methods for the detection and identification of *Leishmania* DNA within the sand flies, which were collected indoors.

*Sergentomyia simillima*, *S*. *ingrami*, and *S*. *africana africana* were found to have fed on humans and, significantly, one pool of *S. ingrami* that had fed on human blood was found to be positive for *L. major* DNA. The remaining two positive *S. ingrami* pools comprised material from specimens that did not contain blood in their guts, suggesting that *S. ingrami* may be able to support *L. major* in its midgut after blood meal digestion without the parasite being expelled during defecation. The identification in the present study of *Leishmania* DNA in human-feeding *S. ingrami* in the same CL outbreak area where the parasite was identified from a CL patient [3] fulfils two essential requirements for the incrimination of a sand fly vector [13], as it provides evidence of a natural *Leishmania*-*S. ingrami* relationship and a possible role of *S. ingrami* as a vector of the disease. However, the presence of parasite DNA alone does not indicate viability of the parasite and further research is required.

Studies conducted in other areas endemic for CL have demonstrated that *Sergentomyia* species such as *Sergentomyia garnhami*, in Kenya [10], *Sergentomyia babu*, in India [9], *Sergentomyia sintoni*, in Iran [35], and *Sergentomyia darlingi*, in Mali [22], can be naturally infected by mammalian *Leishmania*. Human-biting *Sergentomyia* species, *Sergentomyia schwetzi*, *Sergentomyia garnhami*, *Sergentomyia sintoni*, *Sergentomyia clydei*, *Sergentomyia adleri* [1, 15] and more recently, *Sergentomyia dubia*, *Sergentomyia ingrami *, *Sergentomyia simillima*, *Sergentomyia africana africana* and *Sergentomyia ghesquierei* [2, 23, 37], have also been reported in leishmaniasis endemic areas. However, it is important to note that the only species of the genus *Sergentomyia* that has been colonized and with which experimental infections have been conducted, *S. schwetzi*, was refractory to the human-infecting* Leishmania* species *Leishmania major*, *Leishmania donovani* and *Leishmania infantum* [38–40]. The parasites did not complete development in the sand flies and the infections were lost through the defecation of undigested blood remnants [38–40]. It is necessary to establish colonies of *Sergentomyia* species and conduct infection experiments to determine their susceptibility to *Leishmania* parasites and establish their role in the parasites’ transmission in Ghana. Further knowledge of sand fly species is essential to elucidate the transmission dynamics of leishmaniasis and develop effective control strategies for the disease [32].

There were two limitations to this study. First, the focus was on female sand flies, and the males were discarded. Future studies should thus include male sand flies to enable a better understanding of the sand fly population in Ghana. Secondly, an ELISA method was used for the blood meal analysis due to limitations in funding at the time of the study that prevented the use of newer but less easily accessed molecular and matrix-assisted laser desorption/ionization–time of flight protein profiling methods [41–43] which would have increased the accuracy of the findings. In addition, the sampling was conducted more than 15 years ago. Nonetheless, it is important to publish these results as they serve as a baseline for more recent research on CL undertaken in Ghana.

## Conclusions

Neither *Phlebotomus duboscqi* nor *Phlebotomus rhodhaini*, two species of *Phlebotomus* previously recorded in low densities in the study area [3], were detected in the present study, despite the fact that species of the genus *Phlebotomus* are the only reported vectors of leishmaniasis in the Old World [44]. In contrast, the present study shows that some *Sergentomyia* species feed on humans, can harbor *Leishmania,* and could be involved in the transmission of CL in the outbreak area. The observations from the entomological sampling conducted in the outbreak area from 2004 to 2008 strengthen the argument that *Sergentomyia* species may play an important role in the transmission of leishmaniasis in the outbreak focus in Ghana, and add to the growing body of evidence that some *Sergentomyia* species exhibit human-feeding behavior and may be involved in the transmission of CL. To conclusively validate the role of *S. ingrami* in the transmission of leishmaniasis in Ghana, it is necessary to isolate *Leishmania* from *S. ingrami* by dissection and to undertake experimental transmission studies.

## Supplementary Information

**Additional file 1: Figure S1.** Female *Sergentomyia ingrami*. A: pharynx; B: pharyngeal armature; C: cibarium; D: spermatheca (all arrowed).

**Additional file 2: Table S1.** Monthly collection of sand flies using sticky and CDC light traps from January 2006 to May 2007.

## Data Availability

All the data collected during the outbreak investigation were analyzed and are included in the manuscript. Raw data are available on request.
