# Supplementary figures and images for: Investigating potential sand fly vectors after the first reported outbreak of cutaneous leishmaniasis in Ghana
Source: Parasit Vectors. 2023 Apr 28;16:154. doi: 10.1186/s13071-023-05767-4 (PMC10148561; doi:10.1186/s13071-023-05767-4)

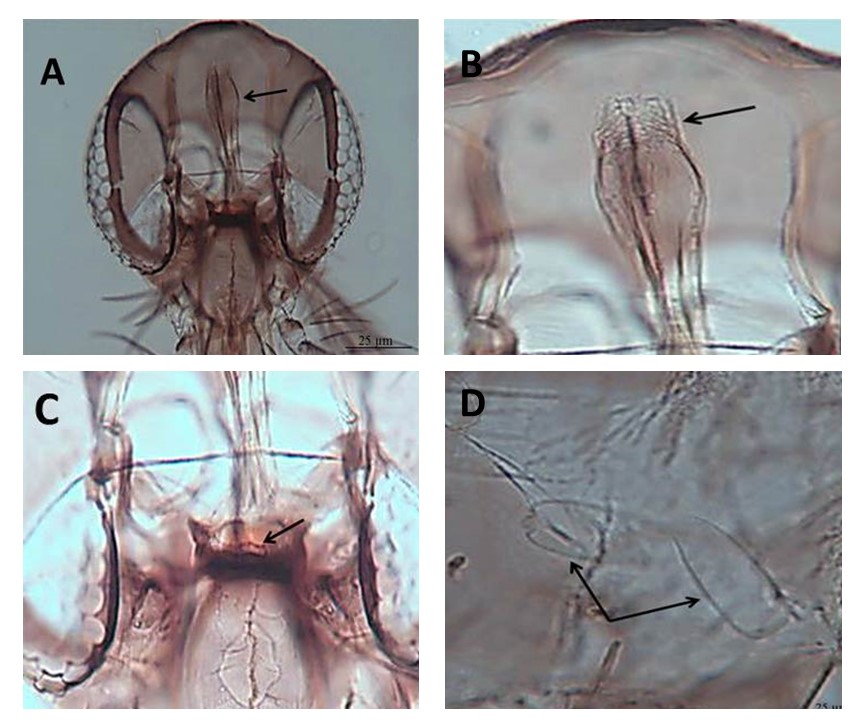

Supplement: Supplementary file 1 — Additional file 1: Figure S1. Female Sergentomyia ingrami. A: pharynx; B: pharyngeal armature; C: cibarium; D: spermatheca (all arrowed). [file 13071_2023_5767_MOESM1_ESM.jpg]
