# Supplementary material for: Investigating potential sand fly vectors after the first reported outbreak of cutaneous leishmaniasis in Ghana
Source: Parasit Vectors. 2023 Apr 28;16:154. doi: 10.1186/s13071-023-05767-4 (PMC10148561; doi:10.1186/s13071-023-05767-4)
Supplement: Supplementary file 2 — Additional file 2: Table S1. Monthly collection of sand flies using sticky and CDC light traps from January 2006 to May 2007. [file 13071_2023_5767_MOESM2_ESM.docx]

Table S1: Monthly collection of sandflies using sticky and CDC light traps from January 2006 to May 2007

|  | Jan 06 | Feb 06 | Mar 06 | Apr 06 | May 06 | Jun 06 | Jul 06 | Aug 06 | Sep 06 | Oct 06 | Nov 06 | Dec 06 | Jan 07 | Feb 07 | Mar 07 | Apr 07 | May 07 | **Total** |
| --- | --- | --- | --- | --- | --- | --- | --- | --- | --- | --- | --- | --- | --- | --- | --- | --- | --- | --- |
| *S. africana* | 93 | 146 | 156 | 344 | 167 | 255 | 135 | 175 | 109 | 145 | 166 | 247 | 230 | 250 | 687 | 523 | 609 | **4437** |
| *S. squamipleuris* | 58 | 107 | 136 | 376 | 227 | 189 | 209 | 232 | 303 | 329 | 102 | 241 | 279 | 2283 | 2445 | 2175 | 1408 | **11099** |
| *S. simillima* | 6 | 8 | 28 | 268 | 117 | 118 | 92 | 192 | 431 | 313 | 476 | 272 | 0 | 16 | 362 | 845 | 599 | **4143** |
| *S. schwetzi* | 13 | 21 | 25 | 73 | 50 | 53 | 48 | 60 | 66 | 19 | 14 | 32 | 12 | 36 | 0 | 0 | 28 | **550** |
| *S. antennata* | 5 | 1 | 11 | 27 | 6 | 13 | 7 | 10 | 0 | 0 | 2 | 5 | 5 | 6 | 17 | 3 | 13 | **131** |
| *S. affinis vorax* | 0 | 0 | 0 | 1 | 0 | 0 | 0 | 0 | 0 | 0 | 0 | 0 | 0 | 0 | 0 | 0 | 0 | **1** |
| *S. pastoriana* | 6 | 0 | 7 | 4 | 0 | 1 | 3 | 3 | 0 | 0 | 0 | 0 | 0 | 0 | 0 | 0 | 0 | **24** |
| *S. hamoni* | 0 | 0 | 8 | 6 | 0 | 0 | 0 | 0 | 0 | 0 | 0 | 0 | 0 | 0 | 0 | 0 | 0 | **14** |
| *S. buxtoni* | 5 | 9 | 9 | 29 | 6 | 2 | 4 | 0 | 5 | 4 | 1 | 0 | 4 | 2 | 4 | 13 | 5 | **102** |
| *S. ingrami* | 1 | 10 | 9 | 10 | 10 | 20 | 2 | 2 | 36 | 72 | 108 | 114 | 50 | 76 | 95 | 57 | 61 | **733** |
| *S. collarti* | 0 | 1 | 3 | 13 | 0 | 6 | 7 | 13 | 11 | 4 | 0 | 0 | 3 | 4 | 7 | 33 | 20 | **125** |
| *S. dissimillima* | 0 | 0 | 0 | 1 | 0 | 0 | 0 | 0 | 0 | 0 | 0 | 0 | 0 | 0 | 0 | 0 | 0 | **1** |
| *S. dubia* | 1 | 0 | 5 | 40 | 2 | 0 | 0 | 0 | 0 | 0 | 0 | 0 | 0 | 0 | 0 | 0 | 0 | **48** |
| *S. bedfordi* | 0 | 0 | 0 | 0 | 0 | 0 | 0 | 0 | 5 | 1 | 2 | 7 | 7 | 0 | 25 | 11 | 40 | **98** |
| *S. dureni* | 0 | 0 | 2 | 9 | 21 | 14 | 0 | 0 | 40 | 39 | 10 | 24 | 43 | 59 | 114 | 66 | 33 | **474** |
| *P. rodhaini* | 0 | 0 | 1 | 1 | 1 | 4 | 1 | 1 | 8 | 1 | 0 | 0 | 2 | 7 | 3 | 3 | 3 | **36** |
| Total | 188 | 303 | 400 | 1202 | 607 | 675 | 508 | 688 | 1014 | 927 | 881 | 942 | 635 | 2739 | 3759 | 3729 | 2819 | **22016** |
